# The contribution of sGAGs to stress-controlled tensile response of posterior porcine sclera

**DOI:** 10.1371/journal.pone.0227856

**Published:** 2020-02-21

**Authors:** Hamed Hatami-Marbini, Mohammad Pachenari

**Affiliations:** Mechanical and Industrial Engineering Department, University of Illinois at Chicago, Chicago, Illinois, United States of America; Universidade Federal do Rio de Janeiro, Instituto de Bioquimica Medica, BRAZIL

## Abstract

Despite the significant progress in characterizing mechanical functions of individual scleral extracellular matrix (ECM) components, the biomechanical contribution of sulfated glycosaminoglycans (sGAGs) is still poorly understood. The primary purpose of this study was to determine the possible function of sGAGs in scleral mechanical response by characterizing the tensile behavior of normal and sGAG-depleted samples. We used chondroitinase ABC solution to remove sGAGs from scleral samples that were dissected from posterior porcine eyes. We performed biochemical analyses for assessing the efficacy of sGAG removal protocol. Furthermore, we conducted stress-controlled uniaxial tensile tests to characterize the influence of sGAG removal on mechanical properties of sclera. The tensile behavior of scleral strips right after dissection and after being soaked in buffer was also determined. Biochemical analyses confirmed that 18 hour incubation in 0.125 U/ml Chondroitinase ABC solution removed over 90% of chondroitin and dermatan sGAGs. No significant difference was observed in the thickness/hydration of samples because of enzyme- and buffer-treated samples. Furthermore, it was found that sGAG depletion did not significantly alter the tangent modulus, energy dissipation, and peak strain of posterior scleral strips. It was concluded that sGAGs did not influence the stress-controlled viscoelastic tensile response of sclera.

## 1. Introduction

The sclera is a dense and opaque connective tissue that makes up the outer surface of the eyeball and defines its size and approximate spherical shape. In addition to preventing internal light scattering, necessary for proper vision, the sclera has important role in protecting inner parts of the eye against external insults. For example, it provides the required stability during eye movement caused by extraocular muscles. Furthermore, unique mechanical properties of sclera prevent significant variations in the shape of ocular globe because of intraocular pressure changes. The biomechanical properties of sclera originate from its specific extracellular matrix (ECM).

The sclera ECM is a complex mixture of primarily collagen fibrils, proteoglycans (PGs), and elastin. Each of these components has a significant contribution to the physiological function of the sclera [1]. The collagen and elastin fibers, constituting the majority of the sclera dry weight, were proven to be the primary loading-bearing components. However, the structural roles of PGs and their sulfated glycosaminoglycan (sGAG) side chains have been less studied. PGs were shown to be essential in regulating the movement of various molecules through the ECM [2]. The most abundant sGAGs in the sclera ECM are chondroitin and dermatan sulfates [3, 4]. Although sGAGs constitute a small percentage of the scleral dry weight, they seem to have a critical role in its proper function [1]. For example, loss of chondroitin sulfates was observed in congenital disorders such as nanophthalmos [5]. It has also been suggested that sGAGs may play a role in myopia, the most common cause of impaired vision [6–8]. The biochemical and biomechanical properties of the sclera were suggested to affect the axial length of the eye. Thus, if changes in sGAG content affect mechanical properties of sclera, it could be hypothesized that sGAGs are important in controlling the ocular axial length and refractive state [9–12].

The mechanical function of sGAGs in tissues such as tendon [13–17], ligament [18, 19], and articular cartilage [20–22] has been extensively examined; however, their possible effects on mechanical properties of scleral tissue have been less studied [23–25]. Furthermore, pressure-controlled inflation experiments showed that sGAG removal had different effects on human porcine samples, i.e. it decreased the stiffness of porcine posterior sclera while it increased the stiffness of human sclera [24, 25]. This contradictory effects of sGAGs have been associated to the older age of human donor samples compared to the porcine ones. However, it is noted that a) an improved inflation technique was used in the later study on human samples in order to measure smaller displacements more accurately [26], and b) there was no direct mechanical comparison between control and enzyme-treated scleral samples.

In general, the inflation testing method investigates the mechanical response of ocular tissues under a physiologically relevant loading condition; thus, it is a preferred technique to characterize the mechanical response of sclera. However, an accurate interpretation of the mechanical measurements is not straightforward. In addition to possible experimental bias errors such as the speckle pattern, image distortion, and illumination condition, the technique is prone to numerical inaccuracies caused by the required inverse numerical analysis for the interpretation of experimental data. In the present study, we took a different approach for characterizing the effects of sGAG digestion on the mechanical behavior of posterior porcine sclera. We enzymatically depleted dermatan and chondroitin sulfates from the posterior porcine scleral strips and characterized their viscoelastic behavior by performing uniaxial stress-controlled tensile experiments. We complemented these mechanical tests by biochemical analyses and hydration/thickness studies.

## 2. Materials and methods

### 2.1 Ethics statement

It is confirmed that this research followed the tenets of the Declaration of Helsinki. This activity does not meet the definition of human subject research and is exempt from IRB review because it only used cadaverous tissue.

### 2.2 Samples

**Fig 1:**
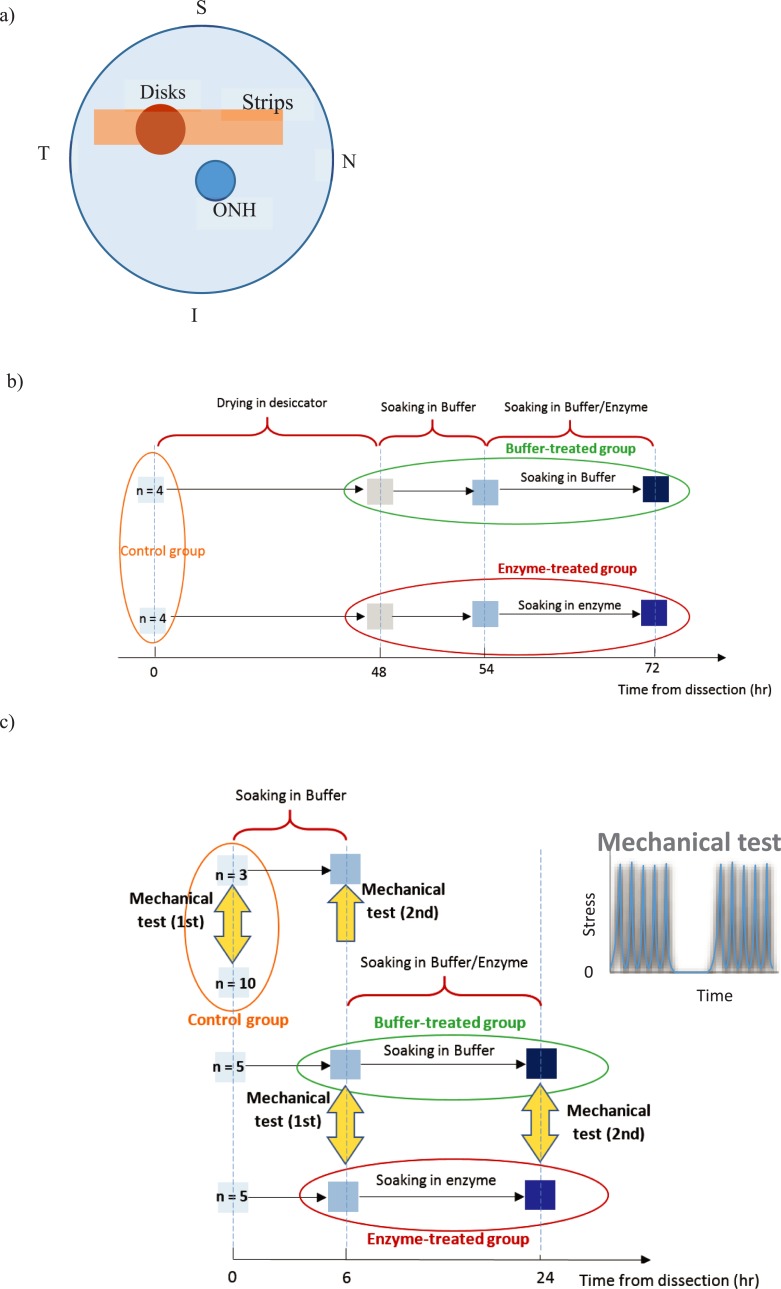
a) Back view of the posterior sclera and the location of excised strips and disks with respect to the optic nerve head (ONH). b) A schematic showing the protocol used to quantify sGAG content of the scleral specimens. c) A schematic showing the mechanical testing protocol and different groups considered in the present study. The samples in the control group were tested immediately after dissection; three of these samples were incubated in buffer for additional 6 hours and tested again. The samples in buffer-treated and enzyme-treated groups were initially soaked in buffer for 6 hours, they were then tested mechanically (first mechanical test), they were then removed from the testing machine and were soaked for 18 hours in either buffer or enzyme solution (while being still mounted to the testing grips), and were then subjected to the second mechanical test. The tensile test protocol, including two five cycles of loading-unloading with a five-minute recovery period in between, is also shown.

Eyeballs of 6 to 8 month old pigs were obtained from a slaughterhouse and tested within 4–6 hours of postmortem. After cleaning the eyeballs and removing their extra muscles/fat, we extracted 25 mm by 4.5 mm scleral strips from the posterior side, Fig 1A. We dissected all strips from the nasal-temporal direction in order to prevent anisotropic bias in the mechanical measurements. We also obtained scleral disks from the superior-temporal quadrant (overlapping with the center of scleral strips), Fig 1A. These scleral disks were solely used for sGAG removal confirmation studies and hydration tests.

### 2.3 Glycosaminoglycan quantification and sGAG removal

sGAGs were digested from the strips/disks by incubating them in 0.125 U/ml Chondroitinase ABC (ChABC) solution for 18 hours at 37 ^o^C. The digestion buffer solution consisted of 50 mM Trizma base, 60 mM sodium acetate at pH 8.0, and 0.02% bovine serum albumin. The Blyscan, i.e. dimethylmethylene blue (DMMB) assay (Biocolor Ltd., UK) was used to determine sGAG content of control (immediately after dissection), buffer-, and enzyme-treated samples, the exact protocol of this assay is well-described in the litrature [27]. Five scleral disks were used for each group and the sGAG content was reported in μg/mg dry tissue weight. The Alcian Blue pH 1.0 Stain Kit (Avantik, New Jersey) was also used for obtaining a qualitative estimate of the success of sGAG removal process. To this end, we stained thin sections of three buffer- and enzyme-treated samples and imaged them later using a whole slide brightfield scanner (Aperio AT2, Leica Biosystems).

### 2.4 Thickness and hydration measurements

The thickness of strips at their centers, near the posterior pole of eyeballs, was measured by a pachymeter (DGH Technology, Inc.). The measurements were averaged over a 2-mm circle in the middle of samples, which approximately coincided with the superior-temporal quadrant. We measured the thickness of scleral disks at their center. A preliminary study confirmed that the pachymeter thickness measurements agreed with those obtained from a micrometer. In order to determine the hydration, we measured the weight of eight scleral discs immediately after dissection and let them completely dry in a desiccator for 48 hours. After measuring their dry weight with 0.01 mg accuracy, we incubated them in buffer solution for 6 hours and measured their wet weight in order to be able to determine their swelling rate. We then immersed the discs either in buffer (*n = 4*) or in enzyme solution (*n = 4*) for 18 hours before measuring their wet weight again, Fig 1B. We computed the hydration of samples by dividing their wet and dry weights.

### 2.5 Mechanical tests

We used an RSA-G2 machine (TA instruments, MD) to characterize the effects of sGAG degradation on the tensile response of scleral strips. The thickness of all strips was measured immediately after dissection and all mechanical experiments were done in buffer solution. In order to prevent possible effects of hydration variation during the mechanical tests [28–30], we soaked all samples in buffer solution for 6 hours prior to the mechanical tests (the hydration study on the scleral disks confirmed that the swelling rate of samples would become very low after 6 hour buffer incubation). The scleral strips were then mounted to the testing machine and their tensile response was measured (these measurements will be referred to as 1st mechanical tests in the following). After the mechanical tests and while they were still clamped (special clamps were designed and machined for this purpose), we removed specimens from the testing machine and incubated them in either enzyme (enzyme-treated group, *n = 5*) or buffer (buffer-treated group, *n = 5*) solution for 18 hours at 37 ^o^C. After measuring the thickness of these incubated strips, we mounted them again to the testing machine and subjected them to the same cyclic tensile load (these measurements will be referred to as 2nd mechanical tests in the following), Fig 1C. The first mechanical measurements of each group represented the tensile properties of samples prior to any significant incubation; these tests allowed us to compare the effects of buffer (or enzyme) treatment on “paired” samples. Comparing the 2nd mechanical measurements of buffer- and enzyme-treated groups together let us determine the effect of sGAG degradation on unpaired specimens.

Thirteen samples (*n = 13*) were also mechanically tested immediately after dissection before being subjected to any significant incubation in any solution; these scleral samples formed the control group and were referred to by symbol C in this work. Three of these samples, after the mechanical tests, were incubated in buffer for 6 hours (without removing them from the grips) to assess possible effects of the 6 hr buffer incubation on the tensile response of paired samples; we used the symbol C-2 to refer to these mechanical measurements in the following, Fig 1C.

We used the destructive DMMB assay on additional five strips to characterize possible variation in the amount of sGAGs during the mechanical tests, i.e. the amount of sGAGs was measured after a strip was a) dissected, b) soaked 6 hours in buffer, c) soaked 6 hours in buffer and mechanically tested, d) soaked 6 hours in buffer, mechanically tested, and soaked in buffer solution for 18 hours, e) soaked 6 hours in buffer, mechanically tested, soaked in buffer solution for 18 hours, and mechanically tested for the 2nd time. We only used one sample per each stage of the testing protocol as incubation in buffer and mechanical tests was not expected to have any significant effect on the sGAG content. Lastly, to confirm that sGAGs were indeed digested from the strips, we determined the sGAGs content of two scleral strips by performing DMMB assay after they were a) soaked 6 hours in buffer, mechanically tested, and soaked in enzyme solution for 18 hours, b) soaked 6 hours in buffer, mechanically tested, soaked in enzyme solution for 18 hours, and mechanically tested for the 2nd time. The experimental *mechanical* measurements for the above scleral strips, i.e. those that their sGAG content was quantified using the DMMB assay, were not used in the mechanical measurement data analysis since they were not subjected to the complete mechanical testing protocol, Fig 1.

In order to have a similar stress history in strips, we included an initial five cycles of loading-unloading with a rate of 1 mm/min and maximum stress of 0.5 MPa in the tensile testing protocol. The preconditioned samples were then allowed to rest for five minutes and their stress-strain behavior was characterized during five cycles of loading-unloading with a rate of 1 mm/min and maximum stress of 0.5 MPa, Fig 1. The second five cycles of loading-unloading were considered in the experimental protocol to be able to confirm that the preconditioning procedure was successful, i.e. the difference between the stress-strain curves obtained from the 5th cycle of the initial loading-unloading step and the 5th cycle of the second loading-unloading step was negligible (results not shown). We only reported here the tangential moduli at 0.05 MPa (low-stress modulus) and 0.4 MPa (high-stress modulus), maximum strain, and hysteresis of the 5th loading-unloading cycle of the second loading-unloading step. Hysteresis, the energy dissipated during one loading-unloading cycle, was calculated from the area within the hysteresis loop. Moreover, we represented the loading curve of the 5th loading-unloading cycle by an exponential relation,

$$\sigma =A\;(e^{B\varepsilon }‐1)$$ (1)

where σ and ε are stress and strain, respectively. The unknown parameters A, B, and C were found by curve-fitting the experimental data. We reported the data as the mean ± standard deviation. We used t-test to test the null hypothesis that the means of two paired groups are equal. Furthermore, we used F-Test to test the null hypothesis that the variances of two unpaired groups are equal. Finally, we used t-Test with equal/unequal variances (determined from the F-Test) to determine whether there is any difference between the means of the two unpaired groups. Finally, we used one-way ANOVA to compare means of several samples when necessary. All statistical analyses were performed with significance level of 0.05.

## 3. Results

Histological images depicted significantly less stain intensity in digested scleral samples (results not shown). The DMMB assay showed that there was more than 90% reduction in the sGAG content because of enzyme treatment. The sGAG content of control (right after digestion), buffer-treated, and enzyme-treated scleral disks was 6.26 ± 1.17, 5.52 ± 1.3, and 0.36 ± 0.33 μg/mg dry tissue, respectively. Furthermore, the sGAG content of different posterior scleral strips from the buffer-treated group, after dissection, after 6 hour buffer incubation, after the first mechanical test, after 18 hour soaking in buffer, and at the end of the second mechanical test, was 5.42 ± 0.92 μg/mg dry tissue (the required dry weight for these five samples was estimated from the hydration study). No significant difference was observed between the sGAG content of either the control group and the buffer-treated group (p = 0.43) or the buffer-treated group and the mechanically tested scleral strips (p = 0.86). This confirmed that multiple buffer incubations and mechanical tests did not cause any significant variation in the sGAG content of specimens. The sGAG content for two strips after 18 hour soaking in enzyme and at the end of the second mechanical test was about 0.39 ± 31 μg/mg dry tissue, i.e. sGAGs were successfully removed from the strips.

Tables 1 and 2 give the hydration and thickness of scleral samples. The hydration of scleral disks was 3.13 ± 0.07, 3.28 ± 0.08, 3.48 ± 0.09, and 3.39 ± 0.10 mg wet weight / mg dry weight immediately after dissection, after 6-hour incubation in buffer, after 18-hour incubation in buffer, and after 18-hour incubation in enzyme solution, respectively. For the samples in the buffer-treated group (Fig 1B), there was a significant difference between the hydration of control samples and those incubated in buffer for 6 hours (P < 0.05). Furthermore, the hydration of samples incubated in buffer for 6 hours was significantly different than the hydration of those incubated in buffer for additional 18 hours (P < 0.05). For the samples in the enzyme-treated group (Fig 1B), there was a significant difference between the hydration of control samples and those incubated in buffer for 6 hours (P < 0.05); however, the hydration of samples incubated in buffer for 6 hours and those incubated in enzyme for additional 18 hours did not vary significantly (P = 0.06). The control (immediately after dissection) samples of buffer-treated and enzyme-treated groups had the same hydration (P = 0.86). Furthermore, there was no significant difference between the hydration of buffer-treated and enzyme-treated groups after 6 hour buffer incubation (P = 0.73) or after 18 hour incubation in buffer/enzyme solution (P = 0.26). The exact same trend, but with different p values, was observed for the thickness variation of scleral disks in buffer-treated and enzyme-treated groups. We did not measure the hydration of mechanically tested scleral strips but their thickness variation was similar to variation of thickness of scleral disks, i.e. the same trend, but with different p values, was observed when calculating the significance of the thickness variation between different strip groups.

**Table 1:** Thickness variations of porcine posterior strips and discs during the experimental protocol, see Fig 1.

Groups	Thickness—Buffer-treated (mm)		Thickness—Enzyme-treated (mm)	
	Sclera strips	Sclera disks	Sclera strips	Sclera disks
**After dissection**	1.21 ± 0.27	1.11 ± 0.10	1.2 ± 0.17	1.08 ± 0.12
**After 6 hr incubation**	1.31 ± 0.32	1.20 ± 0.12	1.32 ± 0.2	1.18 ± 0.13
**After 18 hr incubation**	1.38 ± 0.34	1.26 ± 0.16	1.32 ± 0.16	1.15 ± 0.10

**Table 2:** Hydration, in mg wet tissue weight / mg dry tissue weight, variations of porcine posterior disks during the experimental protocol, see Fig 1.

Groups	Buffer-treated	Enzyme-treated
**After dissection**	3.13 ± 0.07	3.14 ± 0.10
**After 6 hr incubation in buffer**	3.28 ± 0.08	3.25 ± 0.14
**After 18 hr incubation in buffer/enzyme**	3.48 ± 0.09	3.39 ± 0.10

**Fig 2:**
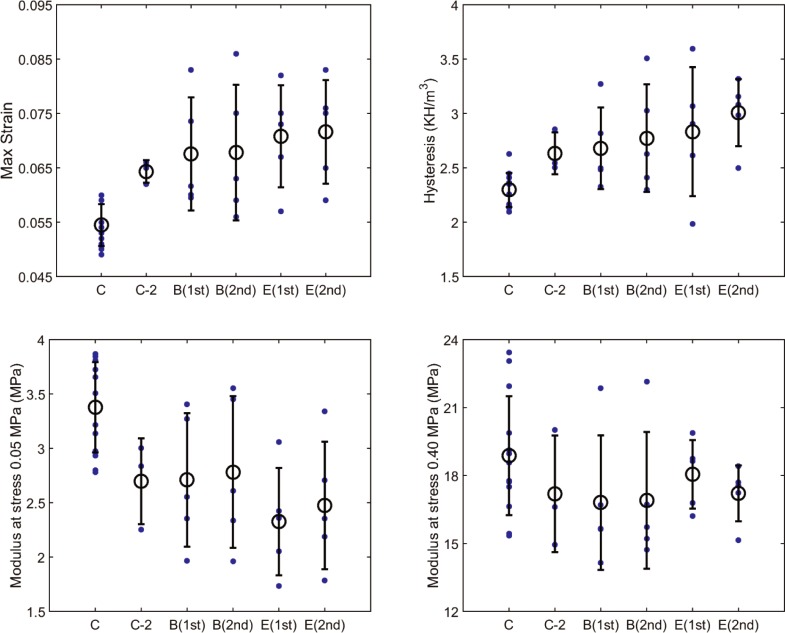
The comparison of mechanical measures that were used to characterize the viscoelastic tensile response of posterior sclera. The measured properties for samples tested immediately after dissection were represented by symbol C and for samples subjected to a second mechanical tested after being tested once and then soaked in buffer for 6 hours by symbol C-2. The measured properties after 6-hour buffer incubation were denoted as B(1st) and E(1st) for strips in the buffer- and enzyme-treated groups, respectively. B(2nd) and E(2nd) represent mechanical properties of the same samples after being incubated for additional 18 hours in buffer and enzyme solution, respectively. The filled circles are the individual data points, the unfilled circles represent the mean values, and error bars denotes one standard deviation.

Fig 2 shows the variation of all four mechanical measures that were used in this study for characterizing the viscoelastic tensile response of buffer and enzyme groups. The samples of the buffer-treated group had maximum strain of 0.068 ± 0.01, modulus at 0.05 MPa of 2.71 ± 0.61 MPa, modulus at 0.4 MPa of 17.00 ± 3.40 MPa, and hysteresis of 2.68 ± 0.375 kJ/m^3^ during the 1st mechanical test. After buffer treatment for 18 hours, the maximum strain, modulus at 0.05 MPa, modulus at 0.4 MPa, and hysteresis of buffer-treated group became 0.068 ± 0.012, 2.782 ± 0.698 MPa, 16.903 ± 3.019 MPa, 2.773 ± 0.495 kJ/m^3^, respectively. No significant difference was observed in any of viscoelastic tensile properties because of the buffer treatment. The samples of the enzyme-treated group had maximum strain of 0.071 ± 0.009, modulus at 0.05 MPa of 2.33 ± 0.5 MPa, modulus at 0.4 MPa of 18.05 ± 1.51 MPa, and hysteresis of 2.83 ± 0.59 kJ/m^3^ during the 1st mechanical test. After enzyme treatment, the maximum strain, modulus at 0.05 MPa, modulus at 0.4 MPa, and hysteresis of enzyme-treated group became 0.072 ± 0.009, 2.47 ± 0.59 MPa, 17.21 ± 1.23 MPa, 3.01 ± 0.31 kJ/m^3^, respectively. No significant difference was observed in viscoelastic tensile properties because of the enzyme treatment. Comparing experimental measurements (i.e. the 2nd mechanical tests) for the buffer- and enzyme-treated samples (unpaired statistical data) showed no significant difference, either. Finally, no significant difference was detected between tensile properties of strips of buffer-treated and enzyme-treated groups during the 1st mechanical tests (unpaired statistical data), suggesting that samples of the present work had the required randomness. Comparing the tensile response of scleral strips that were tested right after dissection (i.e. control group that is represented by the symbol C in Fig 2) with that of strips belong to B(1st) and E(1st) groups, we observed a significant difference in all mechanical measures (P < 0.05) except for the modulus at 0.4 MPa (p = 0.11). This observation suggests that the increase in the hydration of the specimens because the initial 6 hr incubation affected their tensile response. Comparing the tensile response of control group C with that of group C2 showed exactly the same trend with different p values. The difference between these two groups is expected to be because of hydration changes, too.

**Fig 3:**
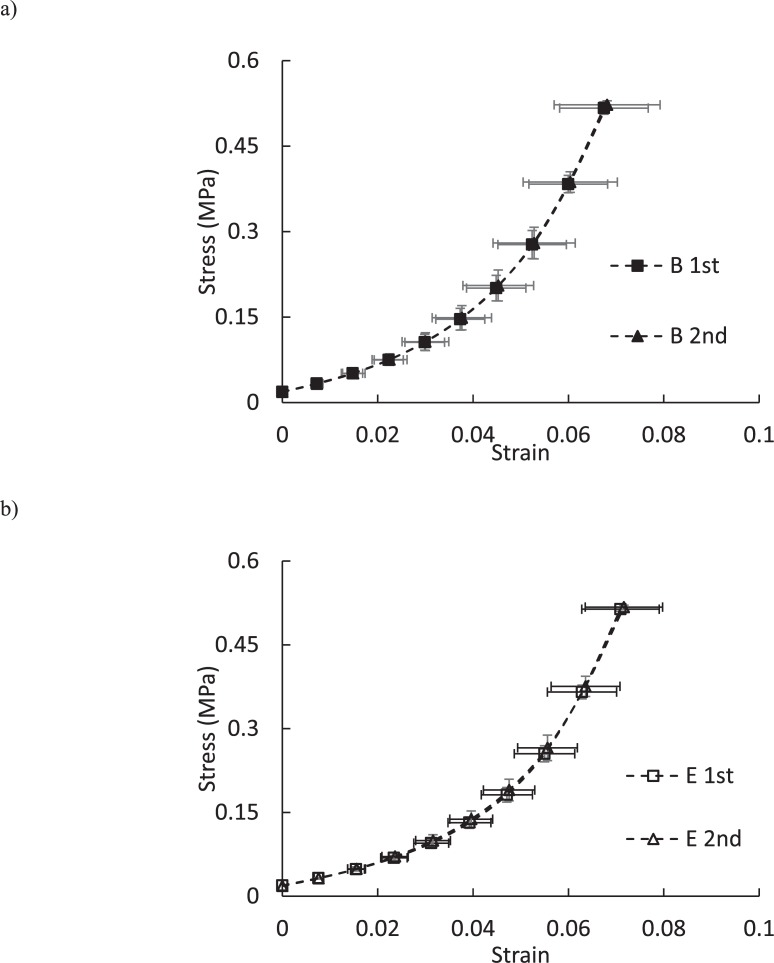
The stress-strain response of a) buffer-treated group and b) enzyme-treated group. The 1st and 2nd refer to tensile properties after 6-hour buffer incubation and additional 18-hour incubation in buffer/enzyme solution, respectively.

Fig 3 shows the stress-strain of posterior scleral strips from the buffer- and enzyme-treated groups. It is seen that neither the buffer treatment nor the enzyme treatment had any significant influence on the stress-strain response of specimens. Furthermore, the exponential expression (1) was able to fit individual experimental measurements with R^2^ > 0.99; the fit parameters are given in Table 3.

**Table 3:** Fit parameters for the exponential function (1), which was used to represent stress-strain behavior of samples during the loading step of the last loading-unloading cycle.

Groups	A (MPa)	B	C (MPa)	R2
**E 1st**	42.1 ± 3.2	0.028 ± 0.008	0.02 ± 0	0.9994 ± 0.0005
**E 2nd**	39.5 ± 2.6	0.034 ± 0.014	0.02 ± 0	0.9995 ± 0.0004
**B 1st**	39.7 ± 9.3	0.043 ± 0.02	0.02 ± 0	0.9997 ± 0.0002
**B 2nd**	38.9 ± 8.6	0.046 ± 0.023	0.02 ± 0	0.9997 ± 0.0002

## 4. Discussion

The extracellular matrix of sclera is composed of elastin, collagen fibers, and PGs. PGs are macromolecule compounds consisting of sulfated chains (sGAGs) that are covalently bonded to a core protein. Both sGAG content of sclera and its viscoelastic mechanical properties have been reported to change in glaucomatous and myopic eyes [7, 31–35]. This suggests that a relation between sGAG content and mechanical response of the sclera might exist. In support of this hypothesis, recent experiments have shown that sGAGs had a significant effect on inflation mechanical behavior of sclera [24, 25]. In particular, it was seen that sGAG removal led to an overall more compliant mechanical behavior of porcine sclera. However, an exact opposite effect was reported later for human posterior sclera, for which the experimental technique was enhanced by improving the lighting and magnification, necessary for capturing smaller displacements more accurately [26]. Furthermore, fewer approximations were used for strain calculation and stress-strain curve analysis, limiting the influence of noise in the data. Although this work concluded that the enzymatic treatment resulted in a significantly stiffer strain-stress response of human sclera (P<0.05), it itself had a few inconsistencies. For example, the data showed that sGAG digestion from the human sclera did not change the strain at maximum pressure (p ~ 1.0). The primary purpose of the present work was to take a more straightforward approach to provide new data on possible roles of sGAGs on mechanical properties of posterior sclera. For this purpose, we determined the mechanical function of sGAGs in the tensile properties of porcine sclera as measured by successive stress-controlled uniaxial tensile experiments. We used chondroitinase ABC for digesting the primary sclera sGAGs, i.e. chondroitin sulfate and dermatan sulfate. We also designed and machined custom built grips in order to be able to characterize possible effects of sGAG digestion on paired samples as well as unpaired groups.

The sGAG content of posterior porcine scleral samples from the present study, i.e. 6.26 ± 1.17, 5.52 ± 1.3, and 0.36 ± 0.33 μg/mg dry tissue for the respective control, buffer-treated, and enzyme-treated samples, was within the same range of those reported by Murienne et al. and Schultz et al. [24, 36]. Schultz et al. reported 4.65 ± 0.33 μg/mg dry tissue weight for control (right after digestion) while Murienne et al. found, respectively, 6.34 ± 1.91, 7.54 ± 1.68, and 1.09 ± 0.23 μg/mg dry tissue for control, buffer-treated, and enzyme-treated porcine samples obtained from the ST quadrant. Murienne et al. reported, without an explanation, a slight increase in the average sGAG content because of buffer-treatment, which disagrees with the present study. The difference between sGAG content of the control and buffer-treated specimens of the present study was not significant (p = 0.43). The slight decrease in the sGAG content due to the buffer incubation was possibly because sGAGs escaped from the samples during the incubation period. We also found that neither the incubation in buffer solution nor the mechanical tests had any significant effect on the sGAG concentration. Furthermore, it was found that the digestion process removed more than 90% of sGAGs from the tissue. The sGAGs were not completely removed primarily because there exist other types of sGAGs, e.g. keratan sulfate, which cannot be digested by chondroitinase ABC. The insufficient incubation time and the lack of complete penetration of digestion solution in specimens might have also played a role. Thus, it is expected that some sulfated sGAG chains were still present in the samples of the present study. However, it is noted the density of these remaining sGAGs was not more than what has been reported in previous sGAG degradation studies of the sclera tissue [24, 25].

The average thickness of control strips of buffer- and enzyme-treated groups was about 1.2 mm, which agreed very well with 1.24 mm that was reported by Murienne et al. [24]. It is noted that we measured the thickness of strips at their center area, which coincides with the thickness measurement of ST samples in Murienne et al.’s work. Similar to this previous study, buffer- and enzyme-treatment caused a significant variation in the mean thickness of strips, when compared to their thickness right after dissection (P < 0.05, paired samples), Table 2. Furthermore, the buffer-treatment after 6 hour incubation significantly increased the thickness (P < 0.05, paired samples) but the enzyme-treatment had an insignificant effect (p = 0.99 for scleral strips and p = 0.3 for scleral disks, paired samples), i.e. sGAG degradation affected the thickness variation of the posterior sclera after 18 hour incubation. This observation agreed with Murienne et al.’s study on human samples but contradicted with their study on porcine samples (these authors contributed this different behavior to differences in sGAG content of pigs and humans). However, the present work using both paired and unpaired samples showed that, similar to human sclera, porcine scleral thickness decreased following s-GAG degradation. We observed an exactly similar trend for thickness variation of scleral disks and strips after being incubated in buffer/enzyme solution. However, the absolute thickness values of strip and disk specimens were different possibly because a) samples from different animals were used, b) scleral disks were first dried (a required step for obtaining their dry weight), and 3) strips underwent mechanical tests.

We did not measure the hydration of samples, which were used in the mechanical tests. This was mainly because we wanted to avoid the possible influence that drying of samples might have had on experimental measurements. However, we measured the hydration response of scleral disks, which confirmed that a) the hydration (thickness) variation significantly slowed down after 6 hours of incubation in buffer solution, b) the equilibrium hydration was about 3.2 mg wet tissue / mg dry tissue, and c) the thickness of specimens had a direct relation with their hydration. We kept all scleral strips in buffer solution for 6 hours prior to mechanical experiments in order to allow them to reach their equilibrium swelling state, i.e. hydration effects on mechanical measurements were prevented so that the influence of sGAG removal could solely be estimated on tensile properties [28–30, 37]. The effect of hydration on tensile properties of scleral samples can be inferred from comparing the mechanical measures for group C with those of groups C-2, B(1st), and E(1st), Fig 3. Similar to their thickness, the hydration of buffer- and enzyme-treated disks did not show a significant difference (P = 0.30); which was again in contradiction with what was previously reported for porcine sclera but consistent with the behavior of human samples (note: the change in hydration of humans after enzyme-treatment was obtained for a single eye in this pervious study) [24, 25]. The small percentage of sGAGs, present in enzyme-treated disks, possibly continued to bring water in the scleral extracellular matrix; this water absorption caused hydration and thickness to increase during their 18 hour incubation. The alternations in the arrangement of ECM of sGAG-depleted samples might have also played a role.

Figs 2 and 3 show that there was no significant difference between tensile mechanical properties of buffer- and enzyme-treated samples. This observation was in contrast to the roles of sGAGs in mechanical response of porcine sclera in inflation [24]. However, our results agreed more with the work on human sclera in which the inflation experimental technique was improved such that displacements were measured more accurately [25, 26]. In this study, the authors reported the mechanical response by averaging over all specimens and quadrants. Thus, it is unclear how sGAG degradation affected the mechanical response of individual quadrants. They also stated that the stiffening effect of sGAG degradation was modest with some mechanical effects (e.g. meridional hysteresis and maximum strain) being insignificant. Another important limitation of Murienne et al.'s work is the absence of direct comparison between control and enzyme-treated porcine sclera, i.e. the main conclusion was reached by comparing the data obtained for control samples (without any treatment) and enzyme-treated samples, which were first subjected to buffer-treatment. Had they used the method that they later used in their study on human samples, i.e. comparing the measurements obtained after incubation in buffer alone (buffer-treated) with those after subsequent incubation in the enzyme solution (enzyme-treated), a different conclusion would have been reached for the effects of sGAGs on the biomechanical properties of the porcine sclera. Finally, it is noted that it is not unprecedented for sGAGs to play different mechanical roles in different loading conditions. For example, despite their significant effects on the viscoelastic creep response of articular cartilage, the extraction of sGAGs had no significant influence on the tensile stiffness and strength when slow constant-rate tensile load was used [38]. Furthermore, no significant contribution of sGAGs to the viscoelastic tensile behavior of human ligaments was found [18, 19].

**Fig 4:**
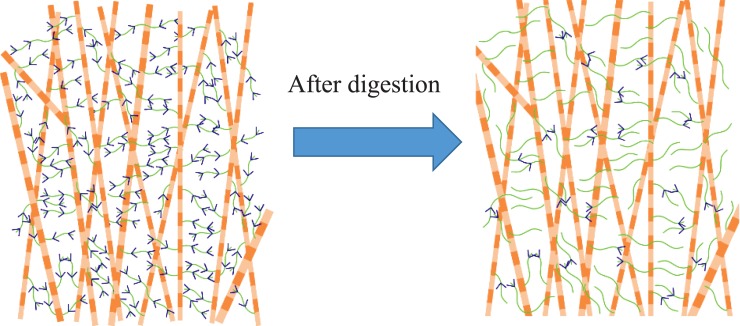
a) The interactions between sGAGs and collagen fiber in the scleral extracellular matrix. b) The collagen fibers are highly interwoven and do not requires sGAGs for load transfer. The remaining sGAGs in enzyme-treated specimens continues to keep the sample hydrated.

The mechanical functions of sGAGs could be explained by considering the extracellular matrix of soft tissues as a composite domain, where collagen fibrils are discontinuous reinforcements that require an interfibrillar matrix, formed by sGAGs and their interaction with each other and collagen fibers, to resist external forces [39, 40]. In this viewpoint, digestion of sGAGs weakens the matrix component causing a softer tensile mechanical response. Another viewpoint is that the main function of sGAGs is to keep the tissue hydrated, and thus, to facilitate the relative movement of collagen fibers [30, 41]. In this model, a stiffer or softer response is predicted depending on whether sGAG digestion reduces or increases the tissue hydration, which in turn increases or decreases the friction between collagen fibers. Here and in agreement with the previous pressure-controlled inflation study [24], the thickness of ST samples did not show any significant variation due to sGAG removal, suggesting that digested samples had similar hydration to buffer-treated samples, Tables 1 and 2. Thus, in agreement with the latter model, the average tensile response of enzyme-treated samples was very similar to that of their corresponding control samples, Fig 3. Unlike the present study, Murriene at al. reported a significantly stiffer response for porcine sclera after enzyme-treatment and attributed it to the significantly higher hydration of these samples. This inconsistency in mechanical roles of sGAGs in porcine sclera requires future investigation focusing on the complex molecular interaction between extracellular constituents. Despite the clear necessity for future research in this area, the present findings suggested that, similar to ligaments and tendons, collagen fibers should be the main load bearing component in the extracellular matrix of the sclera in tension while sGAGs do not play a significant role [13, 15, 18, 19, 42]. This observation is possibly because of very low concentration of sGAGs and the presence of interwoven collagen fibers in sclera, i.e. sGAGs should have a limited contribution to connecting collagen fibers together and transferring loads among them, Fig 4.

The present study used a testing protocol with a preconditioning step; preconditioning of scleral strips might have influenced their viscoelastic tensile response such that no mechanical effects for sGAGs were observed. However, we note that a preconditioning step was also used in the recent inflation study, where a significant role for sGAGs when subjected to pressure loads was observed [24]. Unlike pressure-controlled inflation experiments, uniaxial tensile tests investigate the mechanical response of sclera in a less physiologically relevant loading condition, which is a clear weakness of the present work. However, tensile tests are often used for conducting comparative studies in which the mechanical influence of one parameter is sought [18, 19, 32, 37, 38, 43–45]. The maximum circumferential strain in the previous inflation study was about 0.03–0.06 and 0.003–0.006 for porcine and human samples, respectively. Although the tensile strains of the present study were significantly larger than those of the study on the human sclera, they were comparable with what reported for the porcine sclera under inflation. However, we measured ten times higher stresses, which are in agreement with previous studies that used the tensile testing method [46, 47]. Future studies are required to fully understand why different conclusions were made about the structural roles of sGAGs in tension and inflation. Nevertheless, significantly different testing technique and sample conditions may have contributed to reaching different conclusions.

In summary, this study, for the first time, characterized the contribution of sGAGs to tensile properties of posterior sclera of pig eyes. It was found that sGAG removal had no significant effect on the viscoelastic stress-controlled tensile response as characterized by the tangent moduli, maximum strain, and hysteresis. This was in contrast to the role of sGAGs in the mechanical response of posterior sclera in pressure-controlled inflation. Future studies examining the microstructure of sclera in the absence of sGAGs may be able to provide the required information for explaining these seemingly contradictory conclusions.
